# New tuberculosis vaccines in India: Modelling the potential health and economic impacts of adolescent/adult vaccination with M72/AS01_E_ and BCG-revaccination

**DOI:** 10.1101/2023.02.24.23286406

**Published:** 2023-02-24

**Authors:** Rebecca A Clark, Chathika K Weerasuriya, Allison Portnoy, Christinah Mukandavire, Matthew Quaife, Roel Bakker, Danny Scarponi, Rebecca C Harris, Kirankumar Rade, Sanjay Kumar Mattoo, Dheeraj Tumu, Nicolas A Menzies, Richard G White

**Affiliations:** 1 TB Modelling Group and TB Centre, London School of Hygiene and Tropical Medicine; 2 Centre for the Mathematical Modelling of Infectious Diseases, London School of Hygiene and Tropical Medicine; 3 Department of Infectious Disease Epidemiology, London School of Hygiene and Tropical Medicine; 4 Vaccine Centre, London School of Hygiene and Tropical Medicine; 5 Center for Health Decision Science, Harvard T.H. Chan School of Public Health, Boston, USA; 7 KNCV Tuberculosis Foundation; 8 Sanofi Pasteur, Singapore; 9 World Health Organization, India; 10 Central TB Division, NTEP, MoHFW Govt of India. New Delhi, India; 11 Department of Global Health and Population, Harvard T.H. Chan School of Public Health

## Abstract

**Background:**

India had an estimated 2.9 million tuberculosis cases and 506 thousand deaths in 2021. Novel vaccines effective in adolescents and adults could reduce this burden. M72/AS01_E_ and BCG-revaccination have recently completed Phase IIb trials and estimates of their population-level impact are needed. We estimated the potential health and economic impact of M72/AS01_E_ and BCG-revaccination in India and investigated the impact of variation in vaccine characteristics and delivery strategies.

**Methods:**

We developed an age-stratified compartmental tuberculosis transmission model for India calibrated to country-specific epidemiology. We projected current trends to 2050 assuming no-new-vaccine introduction, and M72/AS01_E_ and BCG-revaccination scenarios over 2025–2050 exploring uncertainty in product characteristics and implementation. We estimated reductions in tuberculosis cases and deaths by each scenario compared to no-new-vaccine introduction, as well as costs and cost-effectiveness from health-system and societal perspectives.

**Results:**

M72/AS01_E_ scenarios were predicted to avert 40% more tuberculosis cases and deaths by 2050 compared to BCG-revaccination scenarios. Cost-effectiveness ratios for M72/AS01_E_ vaccines were around seven times higher than BCG-revaccination, but nearly all scenarios were cost-effective. The estimated average incremental cost was US$190 million for M72/AS01_E_ and US$23 million for BCG-revaccination per year. Sources of uncertainty included whether M72/AS01_E_ was efficacious in uninfected individuals at vaccination, and if BCG-revaccination could prevent disease.

**Conclusions:**

M72/AS01_E_ and BCG-revaccination could be impactful and cost-effective in India. However, there is great uncertainty in impact, especially with varying vaccine characteristics. Greater investment in vaccine development and delivery is needed to raise the probability of success.

## Background

India has the largest global burden of tuberculosis. In 2021, there were an estimated 2.9 million cases and 506 thousand deaths–representing approximately 30% of the total globally.^1^ The COVID-19 pandemic has negatively impacted tuberculosis prevention and care in India, with increases in the number of deaths per year seen for the first time since 2007.^1,2^ Delays in diagnosis and treatment due to surveillance systems impacted by the pandemic (over 30% fewer notifications reported in 2021 than 2019) may lead to increases in the disease burden.^1,2^

Tuberculosis is a key focus for the Indian government. The National Strategic Plan to End Tuberculosis in India 2020–2025, developed by the National Tuberculosis Elimination Programme (NTEP), outlines ambitious goals for reducing *Mycobacterium tuberculosis* (*Mtb*) transmission, preventing tuberculosis disease, and addressing social determinants of health.^3^ Despite the COVID-19 pandemic, the NTEP has made progress toward these goals, including expanding molecular diagnostics, implementing tuberculosis-COVID bidirectional screening, and expanding policy on preventive therapy to include all household contacts of people diagnosed with pulmonary tuberculosis.^4^

The National Strategic Plan also calls for further development in tuberculosis vaccines, which has been a high priority for global organisations such as the World Health Organization (WHO). A recently completed WHO-commissioned study assessing the full value of tuberculosis vaccines made a strong case from the health and economic perspectives for continued investment,^5-9^ and previous work has demonstrated that novel vaccines or vaccination strategies will be needed to eliminate tuberculosis.^10,11^

Currently, sixteen candidates are in various phases throughout the vaccine pipeline, being trialled in a variety of ages and spanning prevention of disease, infection, and recurrence endpoints.^12^ A phase IIb trial of M72/AS01_E_ in adolescents and adults infected with *Mtb* demonstrated a prevention of disease efficacy of 49.7% (95% confidence interval: 2.1–74.2) after three years follow-up.^13^ However, M72/AS01_E_ would need a supportive Phase III trial for licensure, which is planned but likely to require years before results are available to inform policy. Revaccination of uninfected adolescents with the Bacillus Calmette–Guérin (BCG) vaccine was assessed as a third parallel arm in a separate Phase IIb trial, and demonstrated an efficacy of 45.4% (6.4–68.1) against sustained infection,^14^ and an additional Phase IIb confirmation trial is underway to verify this finding, with results expected mid-2024.^15^ The original Chingleput BCG vaccination trial reported efficacy of 27% (−8–50) against disease in children and no efficacy in adults.^16^ A re-analysis of trial data restricted to participants with prior BCG vaccination and no tuberculosis disease at the time of vaccination showed a protective efficacy of 36% (11–54) against disease.^17^ As BCG is already-licensed, introducing BCG-revaccination may only require policy change, which could happen quickly.

India is arguably the most important country for global tuberculosis elimination, and policy makers require country-specific evidence of the anticipated health, cost, and budget impacts of specific vaccine candidates. As vaccines enter Phase III trials, it is important to predict how variation in vaccine profile and implementation will affect impact to maximise benefits and reduce delays between licensure and delivery. We estimated the potential health and economic impact of M72/AS01_E_ and BCG-revaccination in India and investigated the impact of variation in vaccine characteristics and delivery strategies.

## Methods

### Data

We obtained demographic data for India from the United Nations Population Division with estimates for single ages and years from 1900–2100.^18^ Tuberculosis disease and infection prevalence estimates were derived from the National TB Prevalence Survey in India 2019–2021.^19^ Incidence, notifications, and mortality estimates were obtained from WHO.^2^

### Structure

We adapted previous models and developed a compartmental dynamic model of tuberculosis in India.^5,11,20^ Our model was stratified by tuberculosis natural history and treatment, differences in access-to-care, vaccination, and age. We represented tuberculosis natural history by allowing for *Mtb* infection along a spectrum from uninfected to active clinical disease. We assumed a progressive loss of ability to reactivate following infection, with a monotonic decline in reactivation rates for subsequent latency compartments. Active disease was represented by both subclinical and clinical tuberculosis compartments to align with prevalence survey data.^19^ We accounted for time spent on tuberculosis disease treatment and treatment-related costs. Due to the large contribution of private sector treatment in India, we incorporated differences in treatment mortality and completion probabilities between the public and private sector. Full model structure and parameters are in Supplementary Material sections 1-2.

### Calibration

The model was fit to 19 tuberculosis-related calibration targets: the incidence rate (overall and by age in 2000, 2020, and 2025), mortality rate (overall in 2000, 2020, and 2025), notification rate (overall and by age in 2000 and 2020), disease prevalence (overall and by age in 2015 and 2021), infection prevalence (overall in 2021), the proportion of incident cases with treatment history in 2020, the fraction of subclinical tuberculosis among active tuberculosis in 2020, and the prevalence ratio of active tuberculosis between access-to-care compartments in 2020. We calibrated using the *hmer* R package^21^ to perform history matching with emulation followed by ABC-MCMC until we obtained 1000 parameter sets fitting all targets (further information in Supplementary Material section 3).

### Scenarios

#### i. No-new-vaccine baseline

Assuming current trends continue, we used the calibrated model to project baseline epidemiology to 2050 (the “no-new-vaccine” baseline). We assumed that neonatal BCG vaccination would not be discontinued during the period of our analysis and was not explicitly modelled as its effect is implicitly included in country burden estimates.

#### ii. Vaccine scenarios

Using the calibrated model, we simulated Basecase scenarios over 2025–2050 for each product with characteristics informed by clinical trial data.^13,14^ The Basecase M72/AS01_E_ scenario assumed a 50% efficacy prevention of disease vaccine with 10-years protection, efficacious with any infection status aside from active disease at vaccination. We assumed the vaccine would be introduced in 2030 routinely to those aged 15 and as a campaign for ages 16–34 (80% routine coverage, 70% campaign), with a repeat campaign in 2040. Based on expert advice, the vaccine price was $2.50 per dose, assuming two doses per course.

The Basecase BCG-revaccination scenario assumed a 45% efficacy vaccine to prevent infection with 10-years protection, and efficacious without infection at time of vaccination. We assumed the vaccine would be introduced in 2025 routinely to those aged 10 (with 80% coverage) and as a campaign for ages 11–18 (80% coverage) with repeat campaigns in 2035 and 2045. Based on the average estimated BCG price from UNICEF,^22^ the vaccine price was set at US$0.17 per dose, assuming one dose per course.

One-time vaccine introduction costs for both vaccine products were assumed to be US$2.40 per recipient based on vaccine introduction support from Gavi, the Vaccine Alliance, with a further US$0.11 supply cost per recipient. We assumed a 5% wastage rate. For the cost-effectiveness analysis, both costs and health outcomes were discounted to 2025 (when vaccination began) at 3% per guidelines.^23^

We varied vaccine characteristics and delivery strategies univariately from each Basecase scenario to explore the sensitivity of impact results to changes in vaccine profile and delivery strategies (Table 1).

#### India NTEP scenario:

Considering the India NTEP short-term target decisions, we evaluated an additional scenario. This scenario assumed a vaccine with 40% efficacy to prevent infection with 10-years protection and efficaciousness with no current infection at vaccination. This vaccine was assumed to be introduced at the start of 2023 routinely to age 18 and as a campaign for ages 19 and above, with scale up to 80% vaccine coverage over two years.

### Outcomes

We estimated the cumulative number of tuberculosis cases and deaths averted between vaccine introduction and 2050 for each scenario compared to the predicted numbers in the no-new-vaccine baseline. For the India NTEP scenario, we calculated the same health impact outcomes as in the original analysis for 2025 and 2030.

We estimated the annual incremental costs of diagnosis, treatment, and vaccination for each scenario, as compared to the no-new-vaccine baseline in 2020 US$. We calculated the difference in total disability-adjusted life years (DALYs) from vaccine introduction to 2050 for each scenario compared to the no-new-vaccine baseline, using the disability weight for tuberculosis disease from the Global Burden of Disease 2019 study,^24^ and country- and age-specific life expectancy estimates from the United Nations Development Programme.^25^

We calculated incremental cost-effectiveness ratios and 95% uncertainty intervals from the health-system and societal perspectives for each vaccination scenario compared to the no-new-vaccine baseline, calculated as the ratio of mean incremental costs to mean incremental benefits in DALYs averted, for the analytic period 2025–2050. Higher cost-effectiveness ratios indicate greater spending is needed to achieve health improvements, such that the intervention is less likely to be cost-effective. We measured cost-effectiveness by 2050 against three India-specific cost thresholds: 1x gross domestic product (GDP) per capita (US$1,927.71),^26^ and country-level opportunity cost thresholds defined by Ochalek et al (Ochalek upper [US$363] and lower [US$264] bounds).^27^

## Results

The baseline model fit all 19 calibration targets with at least 1000 parameter sets. Epidemiological projections from 2020–2050 are in Supplementary Material section 7.

**Figure 1:**
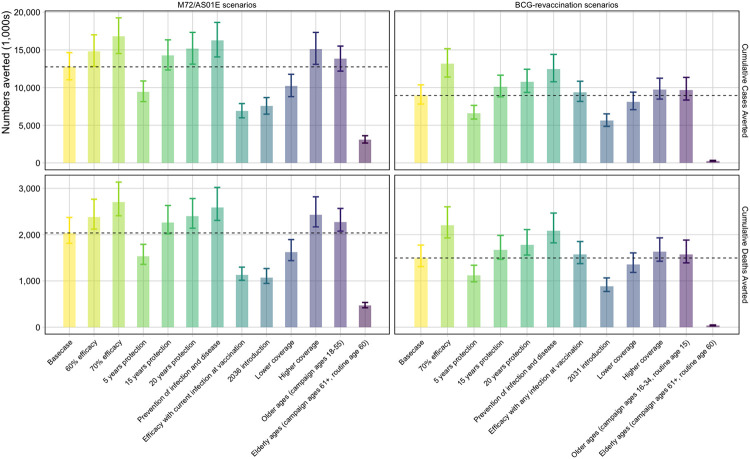
Cumulative cases and deaths averted (in 1,000s) by 2050 from M72/AS01_E_ and BCG-revaccination scenarios. The top of the bar is the median estimate of the number averted for each scenario compared to the estimated number predicted by 2050 with the no-new-vaccine baseline with 95% uncertainty range. The horizontal line is the median value of the Basecase for each vaccine.

We found a 50% efficacy M72/AS01_E_ prevention of disease vaccine, efficacious with any infection status, introduced in 2030 routinely to 15-year-olds and as a campaign for ages 16–34, could avert approximately 12.7 (95% uncertainty interval: 11.0–14.6) million cases and 2.0 (1.8–2.4) million deaths between 2030–2050 (Figure 1). With a 70% efficacy vaccine, the number of averted cases and deaths by 2050 could be increased by 32–35%, but delaying introduction of a vaccine until 2036 could lead to 5.2 million more cases and 968 thousand more deaths before 2050 (Figure 1). If the vaccine was only efficacious with current infection at vaccination, 5.8 million fewer cases and 900 thousand fewer deaths could be averted.

A 45% efficacy prevention of infection BCG vaccine, efficacious in those with no current infection, introduced in 2025 as routine vaccination of 10-year-olds and a campaign for ages 11–18 could avert 9.0 (7.8–10.4) million cases and 1.5 (1.3–1.8) million deaths (Figure 1). If the vaccine prevented infection and disease, 3.4 million more cases and 600 thousand more deaths could be averted by 2050. Fewer numbers could be averted with reduced duration of protection, later introduction, lower coverage, or only delivering the vaccine to ages 60 years and older (Figure 1).

Comparing the two products, we found a higher health impact from M72/AS01_E_ vaccines compared to BCG-revaccination. The Basecase M72/AS01_E_ scenario was predicted to avert around 40% more tuberculosis cases and deaths before 2050 than the Basecase BCG-revaccination scenario. Health impact values for all scenarios of both vaccines are in Supplementary Material section 8.

### India NTEP scenario:

Administering a vaccine with 40% efficacy to prevent infection in those with no current infection at the time of vaccination to 80% of adults aged 18+ over 2 years from 2023 could avert approximately 1.4 (1.2–1.7) million cases and 160 (140–192) thousand deaths by 2030 and reduce incidence and mortality rates by 8–10%. Full health impact results are in Supplementary Material section 8.

**Figure 2:**
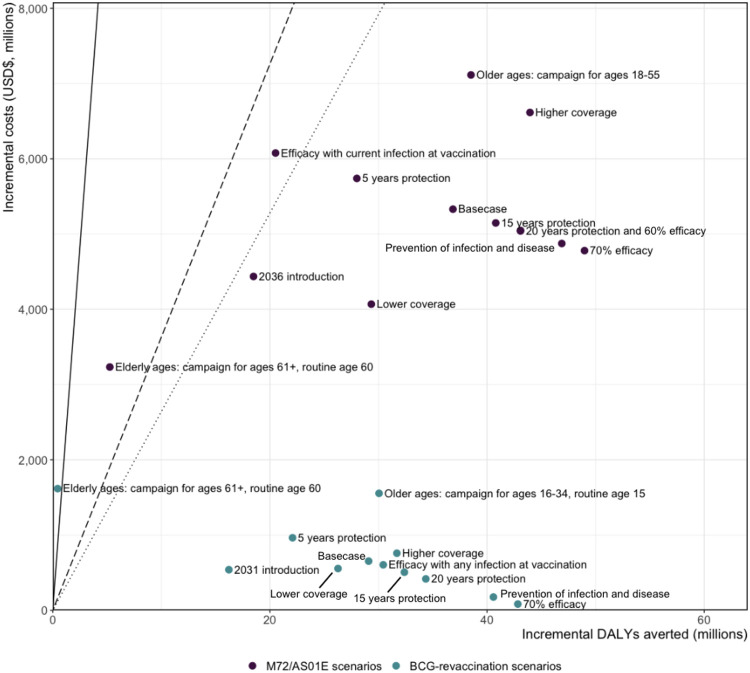
Incremental cost-effectiveness ratios for each scenario compared to the no-new-vaccine baseline. Abbreviations: DALYs = disability-adjusted life years, USD$ = United States dollars. Points are the mean incremental costs and mean incremental DALYs averted for each scenario compared to the costs and DALYs from the no-new-vaccine baseline. Lines shown in the figure indicate cost-effectiveness thresholds based on 1x per-capita GDP (solid line), the Ochalek upper bound (dashed line), and the Ochalek lower bound (dotted line). Points lying to the right of a given line indicate that the scenario would be considered cost-effective compared to the no-new-vaccine baseline. The 20 years protection and 60% efficacy scenarios for the M72/AS01_E_ vaccine overlap and appear as one single point on the figure.

The estimated mean number of DALYs averted between 2030–2050 for the Basecase M72/AS01_E_ scenario was 36.9 (32.5–42.9) million (Table 2). Mean incremental costs were estimated at US$5.3 (3.1–8.6) billion from the health-system perspective and US$5.1 (2.9–8.4) billion from the societal perspective (Table 2). The Basecase M72/AS01_E_ scenario was cost-effective at all thresholds (Figure 2). The scenario delivering the vaccine to ages 60+ was on average only cost-effective at 1x per-capita GDP, and the scenario assuming the vaccine was only efficacious with current infection at the time of vaccination was not cost-effective at the Ochalek lower bound (Figure 2). All other M72/AS01_E_ scenarios were cost-effective at all thresholds. The annual average cost of vaccination in the Basecase M72/AS01_E_ scenario was approximately US$251 (170–370) million between 2030–2050. The annual average cost-savings in treatment and diagnostics were US$60 (49–74) million over 2025–2050.

The estimated number of DALYs averted between 2025–2050 for the Basecase BCG-revaccination scenario was 29.1 (25.1–34.6) million (Table 2). Incremental costs were estimated at US$653 (−419–2,179) million from the health-system perspective and US$502 (−563–2,030) million from the societal perspective (Table 2). The Basecase BCG-revaccination scenario was cost-effective at all thresholds (Figure 2). Aside from delivering the vaccine to ages 60+, which was not cost-effective at any threshold, all BCG-revaccination scenarios were cost-effective at all thresholds evaluated (Figure 2). With efficacy increased to 70% or the ability to prevent both infection and disease, BCG-revaccination was predicted to be cost-saving from the societal perspective (Table 2). The annual average cost of vaccination in the Basecase BCG-revaccination scenario was US$67 (30–122) million over 2025–2050. The annual average cost-savings in treatment and diagnostics were US$43 (35–55) million over 2025–2050.

Compared to the no-new-vaccine baseline, we found that the M72/AS01_E_ scenarios had cost-effectiveness ratios around seven times higher than BCG-revaccination scenarios. However, both Basecase scenarios were highly cost-effective at all thresholds evaluated, and the majority of scenarios for both vaccines were cost-effective at the Ochalek lower bound.

The average annual cost of vaccination in the Basecase M72/AS01_E_ scenario (US$251 million) was almost four times greater than the average annual cost of vaccination with the Basecase BCG-revaccination scenario (US$67 million). Accounting for cost-savings, the average annual incremental program cost in the Basecase M72/AS01_E_ scenario (US$190 million) was over eight times greater than the average annual incremental program cost with the Basecase BCG-revaccination scenario (US$23 million).

**Figure 3:**
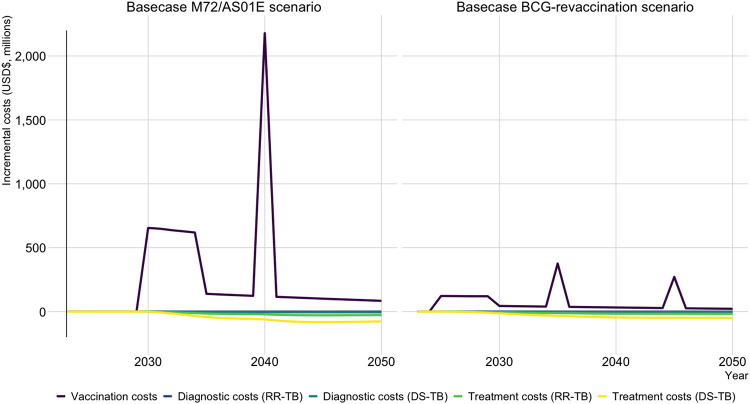
Incremental costs by year until 2050 for the Basecase M72/AS01_E_ and BCG-revaccination scenarios compared to the no-new-vaccine baseline. Abbreviations: DS-TB = drug-susceptible tuberculosis, RR-TB = rifampicin-resistant tuberculosis, USD$ = United States dollars.

Figure 3 demonstrates the distribution of costs and cost-savings per year from vaccine introduction to 2050 for both Basecase scenarios. During the initial 5-year scale-up to target coverage, the vaccination cost for the Basecase M72/AS01_E_ scenario was around US$638 million per year, compared to US$121 million per year for the Basecase BCG-revaccination scenario. The cost of the repeat campaign in 2040 for the Basecase M72/AS01_E_ vaccine was US$2.2 billion, compared to US$376 million and US$271 million, respectively, for the two repeat campaigns in 2035 and 2045 for the Basecase BCG-revaccination scenario. Full economic results are in Supplementary Material section 9.

## Discussion

We found that M72/AS01_E_ scenarios could avert up to 19.3 million cases and 3.1 million deaths, and BCG-revaccination scenarios could avert up to 15.2 million cases and 2.6 million deaths by 2050. Cost-effectiveness ratios for M72/AS01_E_ scenarios were around seven times higher than those for BCG-revaccination scenarios, but nearly all scenarios were cost-effective at the most conservative threshold compared to the no-new-vaccine baseline. The average annual cost of M72/AS01_E_ vaccination was four times greater than BCG-revaccination. Vaccination may lead to an annual incremental program cost of US$190 million for M72/AS01_E_ and US$23 million for BCG-revaccination, accounting for vaccine costs as well as cost-savings.

Our modelling demonstrated a 40% greater health impact from M72/AS01_E_ compared to BCG-revaccination. The difference in impact was due to assumptions made on vaccine characteristics and delivery. Based on clinical trial data and expert opinion, we assumed the Basecase M72/AS01_E_ vaccine would prevent disease and be efficacious in everyone without active disease at vaccination. In contrast, based on trial data,^14,28^ we assumed the Basecase BCG-revaccination scenario would be efficacious only in people without infection at the time of vaccination, and would prevent infection. Therefore, M72/AS01_E_ would be effective in a larger proportion of the population compared to BCG-revaccination and have a more rapid impact on tuberculosis incidence. The effect of BCG-revaccination on disease will be delayed by the time between vaccination and infection in addition to the time from infection to disease. This is consistent with previous work showing more rapid impact on disease of a vaccine that prevents disease directly in those currently infected.^11^

As demonstrated in the National Tuberculosis Prevalence Survey, the highest tuberculosis prevalence estimates are found in older adolescents and adults.^19^ The Basecase scenario for M72/AS01_E_ delivered the vaccine routinely to those aged 15 and as a campaign for ages 16–34, as opposed to BCG-revaccination which was targeted routinely to those aged 10 and a campaign for ages 11–18. As the M72/AS01_E_ vaccine was targeted to an age group with a higher burden, we saw increased impact on burden.

The majority of M72/AS01_E_ and BCG-revaccination scenarios were cost-effective at the Ochalek lower bound. M72/AS01_E_ scenarios were also predicted to have higher vaccination costs per year compared to BCG-revaccination. The assumed M72/AS01_E_ vaccine price per course of US$5.00 (2 doses for US$2.50 each) was almost 30 times the US$0.17 price per course of BCG-revaccination, directly contributing to higher cost-effectiveness ratios and larger annual cost for M72/AS01_E_. Our analyses demonstrated that both vaccines could be cost-effective, aligning with previous cost-effectiveness analyses of tuberculosis vaccines.^6,29^ While vaccination could have a substantial budget impact, costs could be partially offset with diagnostic and treatment savings.

We varied product and implementation characteristics univariately from each Basecase to explore variation in vaccine profile and decisions regarding delivery, and found all uncertainties had the anticipated direction of effect. Both M72/AS01_E_ and BCG-revaccination were highly influenced by vaccine efficacy and duration of protection, with higher efficacies and longer durations of protection increasing health impact and cost-effectiveness. Key sources of uncertainty were whether M72/AS01_E_ was efficacious without infection at vaccination, and if BCG-revaccination was also able to prevent disease in adults, both of which are key areas of research. Given the uncertainty surrounding prevention of disease efficacy from BCG-revaccination, any roll out of BCG to adolescents and adults should be rigorously evaluated with a prevention of disease outcome.

Considering the India NTEP targets, we expanded our analysis to include a scenario aligning with their shorter-term goals. Introducing a vaccine to 80% of the population over 18 years of age, almost 800 million people over two years, could have a considerable impact averting cases and deaths before 2030, even if (as assumed) the vaccine was only efficacious in those with no current infection at the time of vaccination and 40% efficacy. These results can contribute to the evidence used by policy makers to support decisions for vaccine delivery and implementation.

This work has limitations. We modelled M72/AS01_E_ and BCG-revaccination scenarios with characteristics based on clinical trials and expert opinion, but it will be many years before the actual characteristics are known. To capture some uncertainty, we varied efficacy, duration of protection, whether the vaccine prevents only infection or disease or both, and who the vaccine would be efficacious in. The majority of scenarios continued to demonstrate large potential health impact and cost-effectiveness.

We modelled a subset of delivery scenarios, which may differ from the strategies India will choose. We evaluated numerous age-targeting alternatives informed by expert opinion and results from interviews with key decision-makers in India,^30^ but did not investigate targeting specific groups, such as healthcare workers, people completing tuberculosis treatment, or household contacts of people with tuberculosis, who could be at high risk of developing tuberculosis disease and may be prioritised for vaccination. This strategy has previously been suggested to have a high population-level impact per individual vaccinated,^31,32^ and is an important aspect for future country-level models to address, to support decision making.

The burden of tuberculosis varies widely across India. From the recent National Tuberculosis Prevalence Survey, the prevalence per 100,000 population of pulmonary tuberculosis among adults ranged from 115 (47–184) in Kerala to 534 (365–704) in Delhi.^19^ Optimal delivery strategies may vary by state, given the vast differences in age composition, population size, and tuberculosis burden. Modelling specific regions to investigate the generalisability of national predictions is an important area of future research.

Finally, our work is a modelling exercise, and limitations associated with mathematical models apply. We developed our tuberculosis natural history structure incorporating recent advances in knowledge regarding the clinical course of disease, such as subclinical tuberculosis and a latency structure with a progressive loss in the ability to reactivate and made decisions on parameter ranges based on the most recent literature available. If our assumptions around these novel aspects, particularly around interactions with vaccines, are incorrect, we may have over or underestimated the impact.

## Conclusions

We propose it is inadvisable to focus solely on one or two vaccine candidates to address the tuberculosis burden. While promising results have been seen from recent trials, it will be years before we can verify these characteristics, and therefore we need a wide selection of options for the greatest likelihood of mitigating tuberculosis burden. We need to continue investment in all candidates currently in the pipeline, and support the development of new candidates, to increase the probability of success.

Our modelling suggests that M72/AS01_E_ and BCG-revaccination may substantially reduce the tuberculosis burden in India over future decades and would be cost-effective. We informed vaccine characteristics using clinical trial data but found variability in the vaccine profile as a crucial source of uncertainty. We cannot solely rely on M72/AS01_E_ and BCG-revaccination in case the realised characteristics differ considerably from expectations. Investment in multiple vaccine developments and delivery should be increased to raise the probability of success.

## Figures and Tables

**Table 1:** Assumed vaccine characteristics and delivery strategies for M72/AS01_E_ and BCG-revaccination scenarios

	M72/AS01_E_		BCG-revaccination	
Characteristic	Basecase	Varied in univariate	Basecase	Varied in univariate
Vaccine efficacy	50%	60% 70%	45%	70%
Duration of protection	10 years	5 years 15 years 20 years	10 years	5 years 15 years 20 years
Host infection status	AI	CI	NCI	AI
Mechanism of effect	Prevention of disease	Prevention of infection and disease	Prevention of infection	Prevention of infection and disease
Introduction year (Years of any repeat campaigns)	2030 (2040)	2036 (2046)	2025 (2035, 2045)	2031 (2041)
Age targeting	Campaign for ages 16-34, routine age 15	Older ages (campaign for ages 18-55) Elderly ages (campaign for ages 60+, routine age 60)	Campaign for ages 11-18, routine age 10	Older ages (campaign for ages 16-34, routine age 15) Elderly ages: campaign for ages 60+, routine age 60
Target coverage (Scale-up to coverage over 5 years)	Campaign = 70% / Routine = 80%	Campaign = 50% / Routine = 70% Campaign = 90% / Routine = 90%	80%	70% 90%

Abbreviations: AI = any infection, CI = current infection, NCI = no current infection. See Supplementary Material section 4 for full details and references.

**Table 2:** Incremental DALYs averted, incremental costs averted, and ICERs for each scenario compared to the no-new-vaccine baseline.

Scenario	Incremental DALYs averted between 2025– 2050 (millions)	Health-System Perspective		Societal Perspective	
		Incremental costs between 2025–2050 (US$, millions)	ICER (US$/DALY averted)	Incremental costs between 2025–2050 (US$, millions)	ICER (US$/DALY averted)
**M72/AS01_E_ scenarios**					
Basecase	36·9 (32·5–42·9)	5,330 (3,083–8,574)	145 (82–237)	5,121 (2,860–8,375)	139 (77–229)
60% efficacy	43·1 (38·0–50·1)	5,047 (2,807–8,298)	117 (64–196)	4,803 (2,543–8,063)	111 (59–188)
70% efficacy	49·0 (43·2–56·9)	4,780 (2,516–8,063)	98 (50–167)	4,502 (2,235–7,783)	92 (45–160)
5 years protection	28·0 (24·6–32·7)	5,739 (3,507–9,000)	205 (123–326)	5,581 (3,337–8,826)	199 (118–317)
15 years protection	40·8 (36·0–47·4)	5,148 (2,904–8,386)	126 (70–210)	4,916 (2,652–8,174)	120 (65–202)
20 years protection	43·1 (38·0–50·0)	5,042 (2,802–8,293)	117 (64–196)	4,797 (2,536–8,058)	111 (58–188)
Prevention of infection and disease	46·9 (41·3–54·5)	4,875 (2,630–8,145)	104 (55–176)	4,609 (2,343–7,877)	98 (50–170)
Efficacy with current infection at vaccination	20·5 (18·2–23·5)	6,077 (3,856–9,353)	296 (186–456)	5,961 (3,727–9,213)	290 (182–447)
2036 introduction	18·5 (16·2–21·6)	4,437 (2,762–6,877)	240 (145–377)	4,332 (2,656–6,774)	234 (141–368)
Lower coverage	29·3 (25·8–34·2)	4,068 (2,337–6,553)	139 (78–228)	3,902 (2,166–6,398)	133 (74–220)
Higher coverage	43·9 (38·8–51·0)	6,616 (3,852–10,621)	151 (86–245)	6,366 (3,572–10,381)	145 (81–237)
Older ages (campaign for ages 18–55)	38·5 (34·8–43·2)	7,114 (4,177–11,349)	185 (108–294)	6,879 (3,940–11,048)	179 (102–287)
Elderly ages (campaign for ages 61+, routine age 60)	5·3 (4·7–5·9)	3,233 (2,074–4,967)	615 (397–933)	3,183 (2,028–4,889)	606 (387–922)
**BCG-revaccination scenarios**					
Basecase	29·1 (25·1–34·6)	653 (−419–2,179)	22 (cost-saving–79)	502 (−563–2,030)	17 (cost-saving–71)
70% Efficacy	42·8 (37·0–51·0)	82 (−1058–1,684)	2 (cost-saving–40)	−140 (−1,257–1,425)	cost-saving (cost-saving–33)
5 years protection	22·1 (19·0–26·3)	964 (−78–2,456)	44 (cost-saving–116)	851 (−195–2,343)	39 (cost-saving–111)
15 years protection	32·4 (27·9–38·6)	505 (−576–2,049)	16 (cost-saving–67)	336 (−736–1,880)	10 (cost-saving–59)
20 years protection	34·4 (29·7–40·9)	417 (−678–1,970)	12 (cost-saving–60)	237 (−836–1,792)	7 (cost-saving–53)
Prevention of infection and disease	40·6 (35·0–48·4)	175 (−953–1,763)	4 (cost-saving–44)	−35 (−1133–1,526)	cost-saving (cost-saving–38)
Efficacy with any infection status at vaccination	30·4 (26·4–36·1)	604 (−466–2,145)	20 (cost-saving–74)	446 (−628–1,984)	15 (cost-saving–67)
2031 introduction	16·2 (13·9–19·3)	539 (−145–1,522)	33 (cost-saving–97)	456 (−227–1,438)	28 (cost-saving–92)
Lower coverage	26·3 (22·6–31·3)	555 (−390–1,900)	21 (cost-saving–76)	418 (−515–1,764)	16 (cost-saving–69)
Higher coverage	31·7 (27·3–37·7)	758 (−437–2,465)	24 (cost-saving–82)	594 (−591–2,301)	19 (cost-saving–74)
Older ages (campaign for ages 16–34, routine age 15)	30·0 (26·1–35·6)	1,554 (−111–3,953)	52 (cost-saving–135)	1,384 (−301–3,773)	46 (cost-saving–131)
Elderly ages (campaign for ages 61+, routine age 60)	0·4 (0·4–0·6)	1,617 (686–2,997)	3,594 (1,485–6,734)	1,612 (681–2,989)	3,585 (1,478–6,725)

Abbreviations: DALYs = disability-adjusted life years, ICER = incremental cost-effectiveness ratio, US$ = United States dollars. DALYs and incremental costs from both the health-system and societal perspectives were discounted to 2025 (when vaccination began) at 3%. Values in the cells are the mean and 95% uncertainty range.

## List of Abbreviations

BCG: Bacillus Calmette–Guérin
DALY: Disability-adjusted life years
ICER: Incremental cost-effectiveness ratio
*Mtb*: *Mycobacterium tuberculosis*
NTEP: National Tuberculosis Elimination Programme
WHO: World Health Organization
